# Comprehensive Management of Multisite Myiasis in a Critically Ill Patient: A Case Report Highlighting Challenges in Vulnerable Populations

**DOI:** 10.1155/crdi/5574409

**Published:** 2025-12-12

**Authors:** Aryan Shiari, Mohamed Muhanad, Adel Zurob

**Affiliations:** ^1^ Pulmonology, Critical Care, and Sleep Medicine, Mayo Clinic Health System, Eau Claire, Wisconsin, USA, mayoclinichealthsystem.org; ^2^ Infectious Disease, Mayo Clinic Health System, Eau Claire, Wisconsin, USA, mayoclinichealthsystem.org

## Abstract

A 44‐year‐old unhoused male with a history of alcohol dependence was admitted to the emergency department with symptoms of tremors, agitation, and generalized pain. His condition rapidly deteriorated, revealing a subdural hematoma that necessitated an urgent craniotomy. Following surgery, he was diagnosed with multisite myiasis, with maggots present in his ears, nose, eye, and toenail bed. The treatment included surgical removal of the larvae, administration of ivermectin, and broad‐spectrum antibiotics to prevent secondary infections. ENT evaluations confirmed further infestations, identifying the larvae as *Lucilia sericata*. Despite the initial severity, the patient showed significant improvement, regaining full consciousness within a week. This case highlights the critical need for early recognition and comprehensive management of myiasis, especially in vulnerable populations. Effective treatment involves a multidisciplinary approach, including stabilization, surgical intervention, and infection prevention. It underscores the importance of coordinated medical care and preventive strategies to manage myiasis effectively in at‐risk groups.

## 1. Introduction

Myiasis, the infestation by fly larvae, poses significant clinical challenges, particularly in vulnerable populations like the unhoused. Though more common in tropical regions, it can occur worldwide, affecting areas such as the skin, eyes, ears, and nasal passages. Poor hygiene and exposure to flies are key risk factors.

Effective management requires prompt recognition, physical removal of larvae, possibly through surgical intervention, and the use of systemic treatments like ivermectin, along with antibiotics to prevent secondary infections. Preventive measures, including improved hygiene and protective strategies against flies, are essential, especially for at‐risk groups.

## 2. Case Presentation

In September 2024, a 44‐year‐old unhoused male with a history of alcohol dependence was brought to the emergency department by police after being found in a park in Wisconsin, USA. The patient, who had been living in unsanitary conditions, presented with tremors, agitation, and generalized pain. His vital signs were stable: temperature 36.8°C, heart rate 102 bpm, blood pressure 142/85 mmHg, respiratory rate 18 breaths/min, and SpO_2_ 98% on room air. Physical examination revealed a disheveled appearance, ecchymosis of the lower eyelids, and plaque‐like lesions on the scalp. His Glasgow Coma Scale (GCS) score was initially 15 but deteriorated to 7 (E1V2M4) shortly after. A CT scan of the head revealed a subdural hematoma with an 8‐mm midline shift.

**Figure 1:**
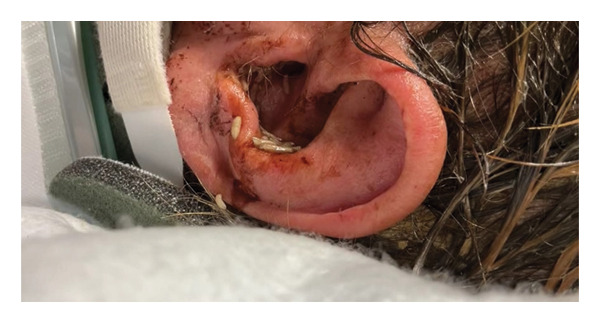
Right ear canal showing active larvae of *Lucilia sericata* prior to removal. The image demonstrates the extent of aural infestation before microscopic extraction.

**Figure 2:**
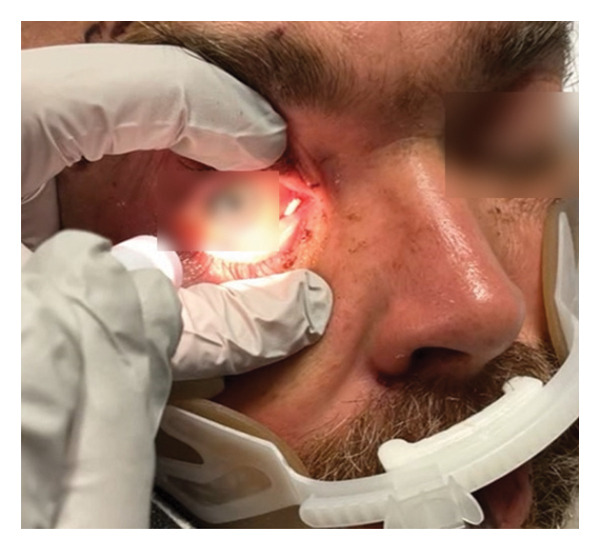
Ophthalmomyiasis externa with visible *Lucilia sericata* larvae along the conjunctival sac prior to removal under topical anesthesia.

**Figure 3:**
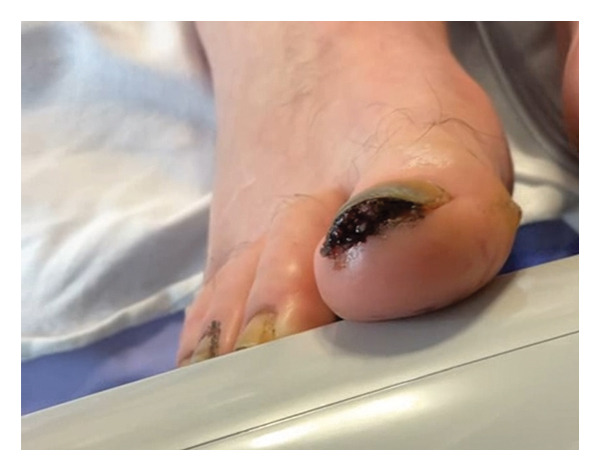
Subungual myiasis involving the right great toenail. Larvae are visible beneath the nail plate prior to extraction.

The patient was intubated and transferred to a tertiary care center, where he underwent craniotomy for the subdural hematoma. He was admitted to the critical care unit (CCU) and treated with standard postcraniotomy protocols, including levetiracetam for seizure prophylaxis and blood pressure management. Following the craniotomy, myiasis was identified, with maggots present in the ears, nasal cavity, right eye, and beneath the right great toenail (Figures 1, 2, and 3; videos 1–3). Given the presence of maggots, the patient received a dose of ivermectin (200 mcg/kg), leading to the exodus of a significant number of larvae. ENT and ophthalmology services performed surgical removal of maggots from the ears, sinuses, and eye. Broad‐spectrum antibiotics (vancomycin, cefepime, and metronidazole) were initiated to prevent secondary infections. Permethrin cream was applied for possible scabies.

A CT sinus scan was performed due to suspicion of sinus involvement from maggots in the nostrils and ears and potential risks of sinus infection and intracranial extension. The CT findings showed mucosal retention cysts and trace air‐fluid levels, with no evidence of worms or significant obstruction. Given the potential small size of larval eggs not easily visualized on CT, a flexible fiberoptic nasolaryngoscopy was completed, revealing larvae in the nasal cavity along with larval eggs. ENT procedures included bilateral examination and removal of maggots from the ears and sinuses, with no maggots found in the ethmoid, sphenoid, or frontal sinuses. Isopropyl alcohol was used to eradicate residual larval eggs.

**Figure 4:**
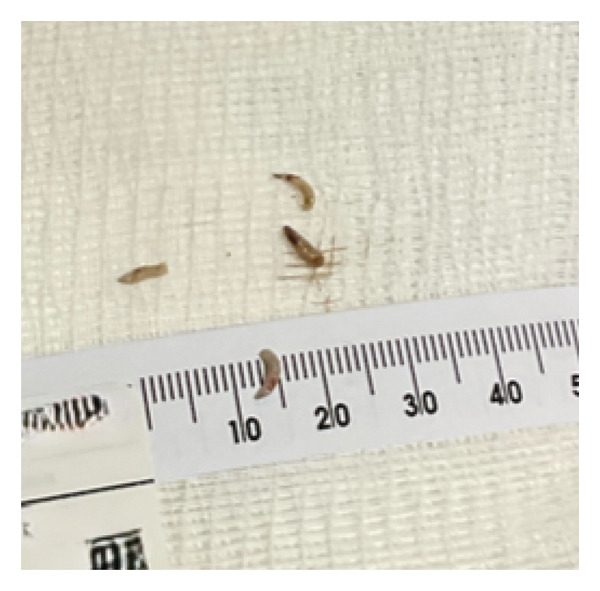
*Lucilia sericata* larvae are pale yellow to grayish and conical in shape (as shown). Eggs are typically white or pale yellow, elongated, and slightly tapered at one end. They are often laid in batches of 150–200 (not shown).

The infectious disease workup included continuation of vancomycin and cefepime to prevent secondary infection, along with ivermectin for myiasis treatment. Larvae extraction was completed for entomological assessment, identifying the organisms as *Lucilia sericata* (Figure 4). HIV screening was negative, and scalp lesion scrapings were negative for fungal organisms. An MRI of the orbit revealed no intraocular involvement.

The patient’s condition improved significantly following treatment. He was extubated within three days, and his GCS improved to 15 within one week of admission.

## 3. Discussion

This case highlights the importance of early recognition and comprehensive treatment of multisite myiasis, particularly in vulnerable populations. Myiasis is the infestation of live human tissue by fly larvae (maggots), which can occur in various sites including ophthalmic, sinus, subungual, and aural regions. Effective management of myiasis indeed involves ensuring patient stability and preventing secondary infections, which are critical care considerations. The global incidence of myiasis is higher in tropical and subtropical regions, with common causative species including *Oestrus ovis* (sheep botfly), *Dermatobia hominis*, *Lucilia sericata*, and *Chrysomyia bezziana* [1–5]. Risk factors for myiasis include exposure to flies, poor hygiene, close contact with livestock, and travel to endemic regions. These factors are well‐documented in the literature, highlighting the importance of maintaining good personal hygiene and taking preventive measures, especially in high‐risk areas [1–5]. On the other hand, maggot debridement therapy (MDT) is an FDA‐approved therapy for certain wound types in the United States. This is done primarily by using sterile *Lucilia sericata* larvae [6, 7].

The distinction between the obligate myiasis (e.g., *Dermatobia hominis*) and opportunistic myiasis (e.g., *Lucilia sericata*) is important for the diagnosis and management of myiasis [8–10]. The obligate species require living tissue; as such they have evolved mechanisms for host invasion, they cause localized furuncular lesions, and the mature larvae are cylindrical, reaching 23 mm in length [9].

The opportunistic species can either develop in living or necrotic tissue, they infest pre‐existing wounds through flexible opportunistic methods, and the larvae are conical ranging from 12 to 18 mm long (Figure 4) [11].

In terms of treatment, the primary approach involves the physical removal of larvae, often under local anesthesia, followed by the application of topical antibiotics to prevent secondary bacterial infections. In more severe cases, systemic treatments such as ivermectin may be used [1–5]. Preventive strategies include educating patients about the importance of wound care, maintaining personal hygiene, and using protective measures against flies, especially in endemic regions. The life cycle of fly larvae in myiasis involves several stages: egg deposition by adult flies, larvae hatching and invading host tissue, feeding and growth within the tissue, and the potential for secondary infections. The host response typically includes an inflammatory reaction, tissue damage, and necrosis.

In the case of ophthalmomyiasis, the condition can be classified into two types based on the degree of involvement: ophthalmomyiasis externa and ophthalmomyiasis interna. Ophthalmomyiasis externa is confined to the ocular surface and periorbital tissues, while ophthalmomyiasis interna involves intraocular penetration.

The patient in this case had ophthalmomyiasis externa, which is commonly caused by species such as *Oestrus ovis*, *Dermatobia hominis*, and *Chrysomya bezziana*. Symptoms often include a foreign body sensation, movement, redness, tearing, itching, swelling, irritation, photophobia, burning, and ocular discharge [12, 13]. The management of ophthalmomyiasis externa and interna involves distinct approaches based on the severity and location of the infestation. For ophthalmomyiasis externa, the primary treatment involves the physical removal of larvae under topical anesthesia, followed by the application of topical antibiotics and corticosteroids to manage inflammation and prevent secondary infection [14]. For ophthalmomyiasis interna, which involves the invasion of the ocular posterior segment, treatment is more complex. The mainstay treatments include laser photocoagulation for subretinal larvae and pars plana vitrectomy for intravitreal larvae removal. Additionally, systemic treatments with ivermectin and steroids have been reported to be effective in severe cases of orbital myiasis, although their use in ophthalmomyiasis interna specifically is less well‐documented [15–17].

Aural and nasal sinus myiasis presents with symptoms such as ear and sinus pain, hearing loss, anosmia, purulent or bloody discharge, tinnitus, vertigo, facial weakness, and neurological manifestations secondary to intracranial involvement. Diagnosis typically involves history and clinical examination, with direct visualization of larvae by ENT specialists. Management includes the removal of larvae under microscopic guidance, irrigation of the ear or sinuses with solutions like alcohol or saline, and broad‐spectrum antibiotics to prevent secondary infections [18, 19]. Previous literature has also documented isolated cases of aural myiasis. Wang et al. described a patient with congenital mental retardation who developed *Lucilia sericata* infestation limited to the external auditory canal, successfully treated with mechanical extraction and antibiotics [20]. In contrast, our case demonstrates simultaneous aural, nasal, ocular, and subungual involvement following neurosurgery in a critically ill patient, requiring a multidisciplinary approach that included systemic ivermectin and surgical extraction.

Subungual myiasis, although rare, can occur in individuals with poor personal hygiene, immunosuppression, or specific conditions like diabetes or peripheral neuropathy. Diagnosis involves detecting larvae under the nail plate, and treatment includes manual removal of larvae, antiseptic footbaths, and systemic antibiotics [21].

The patient’s significant improvement following a multifaceted approach—including stabilization, imaging, rapid surgical intervention, treatment of myiasis, debridement, broad‐spectrum antibiotics, topical treatments, and supportive care—highlights the necessity of timely and coordinated medical intervention. This case reinforces the importance of early recognition and a multidisciplinary approach, to prevent morbidity from myiasis in critically ill or socially vulnerable patients.

## Consent

All the patients allowed personal data processing and informed consent was obtained from all individual participants included in the study.

## Disclosure

The work was conducted as part of the employment of the authors at Mayo Clinic Health System. The funder had no involvement in the manuscript writing, editing, approval, or decision to publish.

## Conflicts of Interest

The authors declare no conflicts of interest.

## Funding

This research and the publication of this article did not receive specific funding from any external sources.

## Supporting Information

Additional supporting information can be found online in the Supporting Information section.

## Supporting information


**Supporting Information 1** Video 1: Visualization of *Lucilia sericata* larvae in the patient’s ear canal, demonstrating aural myiasis.

**Supporting Information 2** Video 2: Visualization of *Lucilia sericata* larvae in the patient’s right eye, illustrating ophthalmomyiasis.

**Supporting Information 3** Video 3: Visualization of *Lucilia sericata* larvae in the patient’s first toenail bed, indicating subungual myiasis.

## Data Availability

Data sharing is not applicable to this article as no datasets were generated or analyzed during the current study.
